# Health selection among outmigrants, return migrants and non-migrants in three subcohorts of international, interprovincial migrants and non-migrants in Manitoba, Canada

**DOI:** 10.23889/ijpds.v9i5.2793

**Published:** 2024-09-18

**Authors:** Katy Huxley, Anna Skeels

**Affiliations:** 1 Cardiff University

Across the UK, the National Referral Mechanism (NRM) is the framework by which those affected, or those at risk of, modern slavery, are referred by ‘first-responders’ (such as the police) to other support services. For children, a key support mechanism is the Independent Child Trafficking Guardianship (ICTG) service, run by Barnardo’s for the Home Office.

Administrative data from the NRM and ICTG were utilised in a novel mixed-method study to evaluate child-centred outcomes for children receiving ICTG support. We utilised publicly available NRM data to look at the nature of reported modern slavery between 2017 and 2022. We also negotiated access to Home Office held ICTG service data for the same period.

Whilst our findings reveal patterns in the extent of reported exploitation and the nature of service support, examination of the two data sets revealed a number of issues related to categorisation of children, exploitation experiences, and geographical representation that hinder a comprehensive understanding of outcomes for child exploitation and trafficking. Qualitative ICTG data, however, revealed a ‘pyramid’ of support areas that were crucial to supporting positive outcomes, whilst participatory research methods revealed significant positive outcomes for children.

The research identified a failure of administrative data to directly collect information relevant to positive child-centred outcomes. The recommendations, detailed within the final report, suggest that there is further work that could be done to improve administrative data collection, and highlight the potential of linked administrative data sources to understand related outcomes, such as health, education and crime/justice.

